# A modular and customizable open-source package for quantum voltage standards operation and control

**DOI:** 10.1371/journal.pone.0209246

**Published:** 2018-12-17

**Authors:** Paolo Durandetto, Andrea Sosso

**Affiliations:** 1 INRiM - Istituto Nazionale di Ricerca Metrologica, Torino, Italy; 2 Politecnico di Torino, Torino, Italy; University of Massachusetts Lowell, UNITED STATES

## Abstract

This paper presents an open-source package developed in Python that controls and drives a programmable Josephson array to synthesize dc and ac quantum-accurate voltages. Programmable arrays are devices subdivided into independent subsections, each counting a number of series connected Josephson junctions that follows a binary sequence (1, 2, 4, 8, …) to control the output voltage. Our software allows to independently measure the current-voltage characteristics of each subsection by means of a set of arbitrary waveform generators and a nanovoltmeter that measures the voltage across the whole array with high sensitivity. A quantization test tool is also provided to check with sub-microvolt resolution whether the array is operating on its quantum margins. The code is modular and easily expandable with the support of many libraries, allowing prompt reconfiguration for different calibration and testing purposes. It is aimed at being a starting point for cooperation between National Metrology Institutes towards the realization of a shared quantum voltage calibration infrastructure.

## Introduction

**Fig 1:**
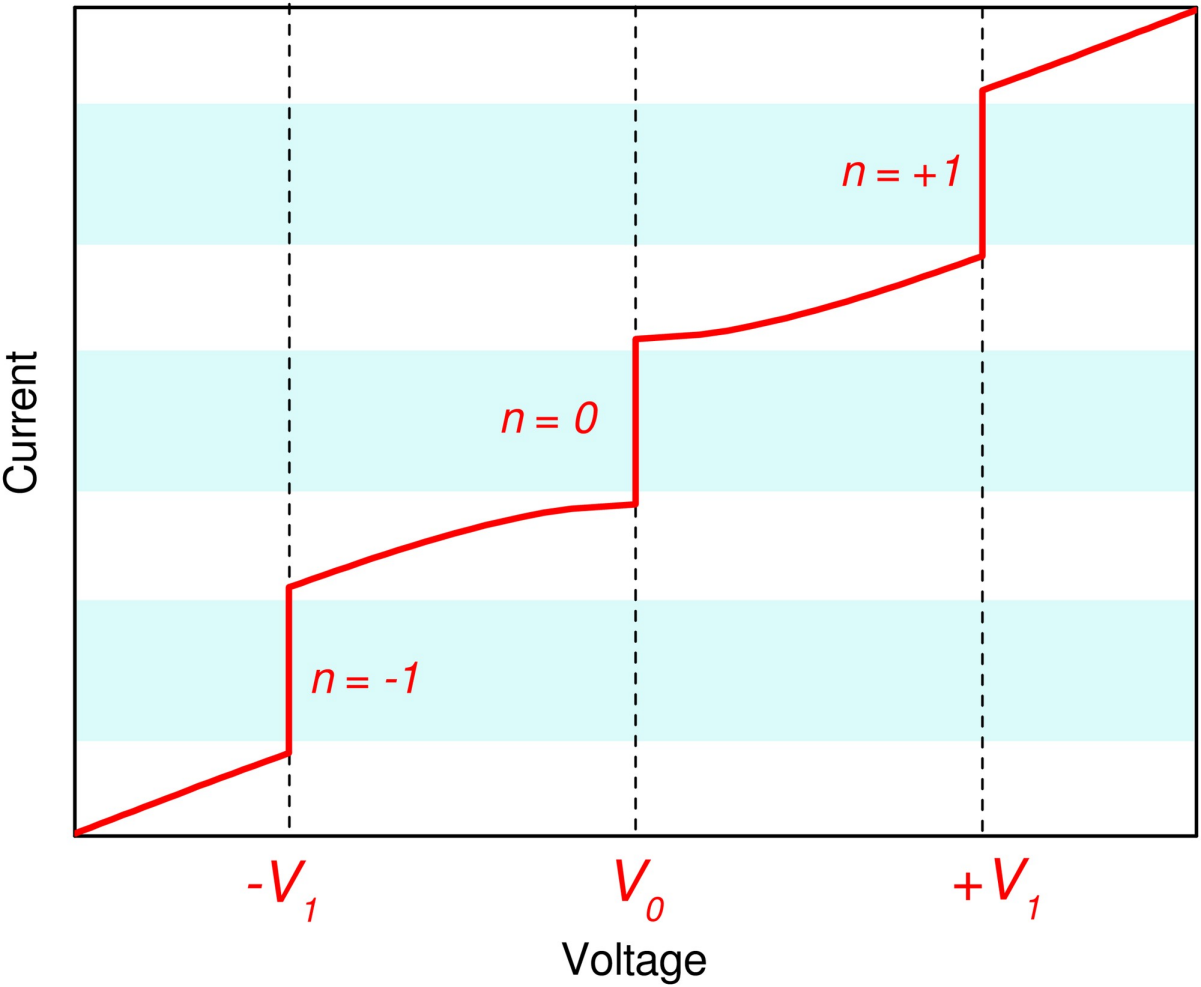
Ideal current to voltage characteristic of a Josephson junction under rf irradiation. Quantized steps of order *n* = 0 and *n* = ±1 are clearly visible, along with their bias currents operating margins (blue shaded area).

The Josephson effect [1] is a macroscopic quantum phenomenon that occurs in devices made with two superconducting elements separated by a thin metallic or insulating layer. These are known as Josephson junctions and find application in many different research fields, from quantum voltage metrology to accurate magnetic flux measurements, from supercomputers to quantum information. In voltage metrology applications, Josephson junctions are operated under the irradiation of an rf-field: this high-frequency excitation causes the appearance of quantum voltage steps in the Josephson junction current-voltage characteristic (Fig 1), known as Shapiro steps [2], whose value is given by:

$$\begin{array}{c} V_{J,n}=\frac{nh}{2e}f_{rf} \end{array}$$ (1)

where *n* = 0, ±1, ±2, … is the Shapiro step order, *h* is Planck constant, *e* is the elementary charge and *f*_*rf*_ is the rf frequency. The amplitude of each step depends on several factors, as junction electrical parameters, rf power and operating temperature.

For a typical rf-field of 70 GHz, the voltage *V*_*J*,1_ across a single Josephson junction is about 145 μV, hence thousands of them are connected in series to attain practical voltages of at least 1 V.

For decades National Metrology Institutes (NMIs) have been taking advantage of the universality of Eq 1 [3] and of the high accuracy of frequency standards (up to 10^−15^) [4] to employ Josephson arrays as primary standards capable of providing dc voltages up to 10 V with accuracy up to 10^−9^ [5].

**Fig 2:**
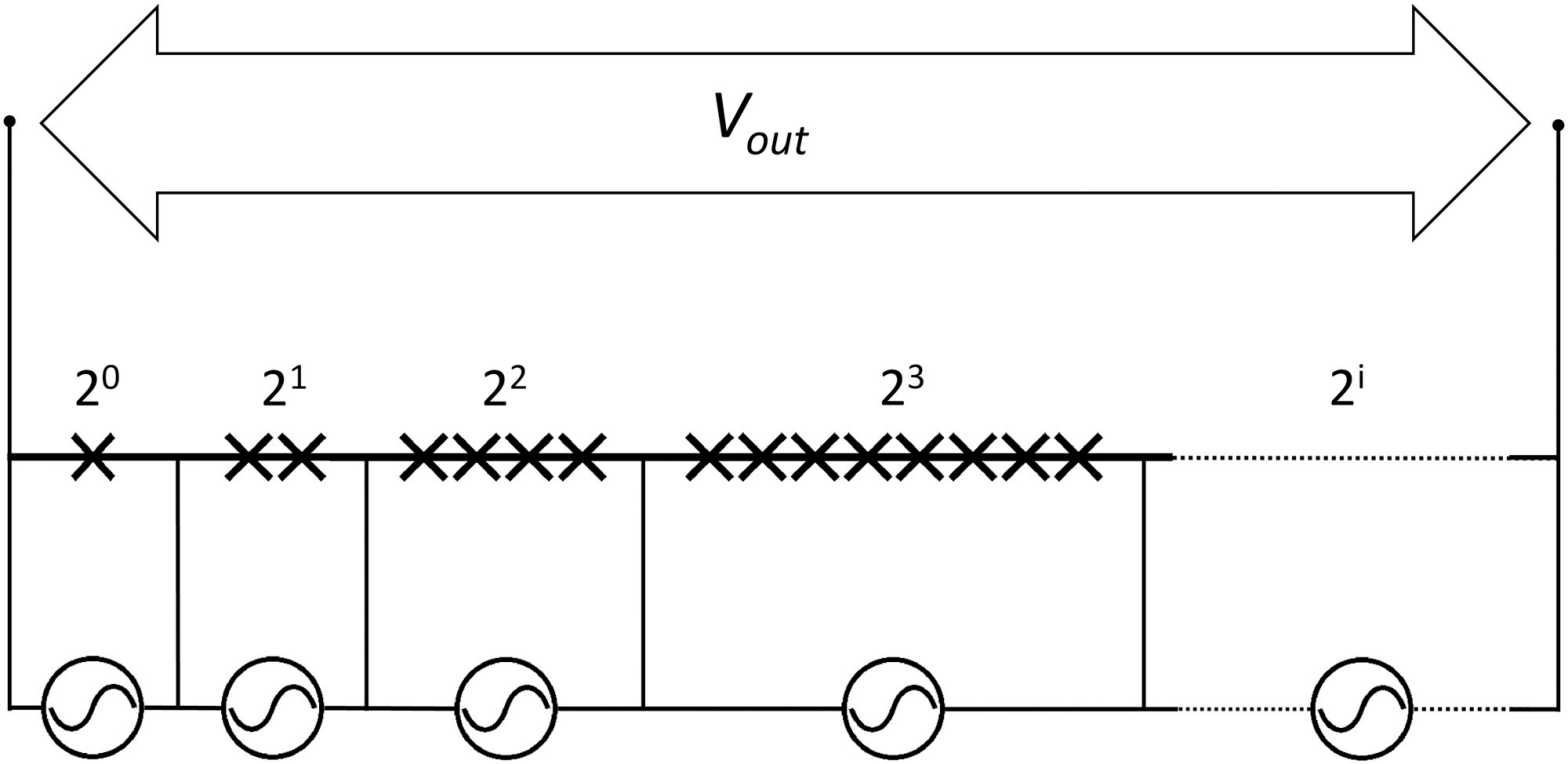
Schematics of a PJVS binary-divided array. A single Josephson junction is represented by ×. Each subsection is individually biased on its *n* = 0 or *n* = ±1 quantum step by adjusting the current *I*_*i*,*n*_.

For the calibration of ac voltages with Josephson devices, to date, the most successful solution is represented by Programmable Josephson Voltage Standards (PJVSs). As shown in Fig 2, a PJVS array is split into subsections, each having a number of junctions equal to a power of two [6].

Following the operating principle of conventional digital-to-analog converters, PJVSs enable the synthesis of stepwise ac voltage signals by properly driving each subsection on its *n* = 0 or *n* = ±1 quantum step, i.e. selectively turning subsections off or on. PJVS devices capable of generating voltage signals up to 20 V have been realized [7], with a frequency limitation of few kHz due to the switching of bias electronics during transients between quantum steps [8].

Nowadays, PJVSs are commercialized by Supracon AG [9, 10] and National Institute of Standards and Technology (NIST) [11] in cutting-edge calibration systems for dc and ac voltages up to 10 V and frequencies up to the kHz range. When employed for calibrating sinusoidal voltages, such systems are usually named ac quantum voltmeters: a fast digitizer samples the difference between an ac voltage signal provided by the instrument under test and a synchronized stepwise-approximated version of the same sine wave generated by the PJVS. The digitizer works as a null detector, thus improving the overall accuracy, and its output is used as a correction term, so that the rms of the waveform under calibration can be accurately calculated from the measured difference samples and the perfectly known reference waveform steps of PJVS [12, 13]. Ac quantum voltmeters allow high-stability ac voltage sources to be calibrated with relative accuracies up to the 10^−7^ level [14]. Proprietary software is distributed as key component of such quantum-based voltage calibration systems: these are generally developed in LabView and are mostly oriented toward calibration activities rather than research needs, where a direct access to the source code is generally preferred. They typically provide the user with a wide variety of functions, as the check of the PJVS electrical characteristic, the automatic search of the optimal operating conditions (microwave power, bias currents) and the most accurate calibration of dc and ac voltages.

Despite the proven success and the promising continuous development of the, more recent, pulse-driven Josephson standards [15, 16], research on PJVS is still ongoing. Furthermore, only few NMIs employ PJVS devices for calibration purposes so far.

The spread among and outside NMIs of measurement capabilities provided by Josephson devices is in fact the central goal of the ACQ-PRO EURAMET project [17]. In this context, software plays a key role, as it represents a main component of the measurement procedure. Yet, most metrologists involved in primary standards research prefer, when possible, to develop their own software as a way of keeping full control of the measurement procedure and total flexibility in the experimental setup [18]. On the other hand, writing code for complex tasks is time-consuming and tricky: in order to avoid replications, increase reliability and speed-up debugging, a standardized code offers great advantages. The development of software for shared use has been recently addressed in quantum voltage metrology. In particular, the need for a shared and coordinated software development is clearly witnessed in the activities planned in several ongoing EURAMET projects [19, 20]. The open-source approach offers a well-known and tested framework for these needs.

Among the most successful open-source results, Python is a programming language widely used for scientific applications. It is an interpreted high-level general-purpose language aimed at code readability, whose syntax allows to minimize lines of code [21]. Python runs on interpreters that are available for almost all operating systems and provides access to a huge and comprehensive scientific library, from signal processing to instrument interfacing.

In the following we present an open-source Python software package for testing and operating PJVS and its applications in the synthesis of quantum-based ac voltage signals. It is then expected to be used by researchers and metrologists with suitable expertises and technical skills in the operation of PJVSs. The program is modular, fully customizable and easily expandable, e.g. to include additional instruments or data processing to provide, for instance, real-time noise and uncertainty analyses. The software is currently in its alpha-stage, hence the users are invited to cooperate with us in its further development and refinement: the source code is publicly available under GNU-GPLv3 license on *GitHub* [22].

## Experimental setup

In its current version, the software is suited for the control of four 4-channels arbitrary voltage waveforms generators (AWGs), Active Technologies AT-AWG 1104 in our implementation (brand names are used for identification purposes, such use implies neither endorsement by INRiM nor assurance that the equipment is the best available) and a nanovoltmeter (Keithley 2182A) to properly drive a 13-bit 1 V PJVS [23] for the synthesis of staircase-approximated quantum voltages and for junctions testing purposes. Employing different instruments is feasible and straightforward just by modifying few functions in the code.

**Fig 3:**
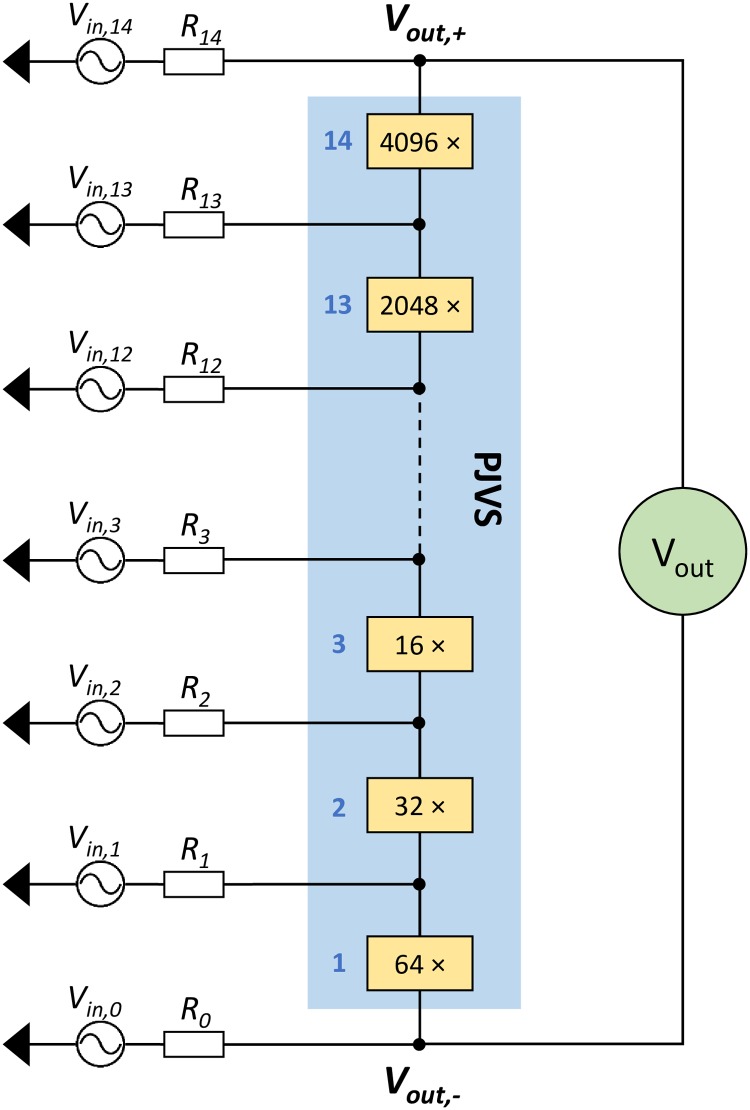
Circuital representation of the 13-bit PJVS system connected to fifteen independent AWG channels (*V*_*in*,*i*_). The blue shaded area shows the PJVS array and the rectangular boxes specify the number of junctions of each subsection. *R*_*i*_ is the output resistance of the *i*^*th*^ AWG channel, which can be selected among 50 Ω, high impedance (open circuit) and low impedance (short circuit) in order to accomplish the desired operating mode.

The 13-bit PJVS in use consists of 8192 Josephson junctions subdivided into fourteen sub-arrays. As shown in Fig 3, the *i*^*th*^ subsection counts *N*_*J*_(*i*) junctions, where *N*_*J*_ = [64, 32, 16, 8, 4, 2, 1, 1, 128, 256, 512, 1024, 2048, 4096] and *i* is an integer number between 1 and 14. The reason of this apparently atypical arrangement is related to the optimal rf transmission along the Josephson array [24]. Fifteen AWG channels (four AT-AWG1104 modules) are required and, in order to exploit all the software potentialities, connections should be done as shown in Fig 3. Two subsections counting a single junction are necessary for having two equal branches, each with 4096 junctions. This feature gains fundamental importance when accurate quantization test is required, as explained in the following. Again, the software is readily customizable in order to comply with PJVS arrays of different structure, as is the case of the 10 V PJVS [25].

## Software description

We describe here the software package from both final user and developer’s points of view.

### User’s point of view: The Graphic User Interface

**Fig 4:**
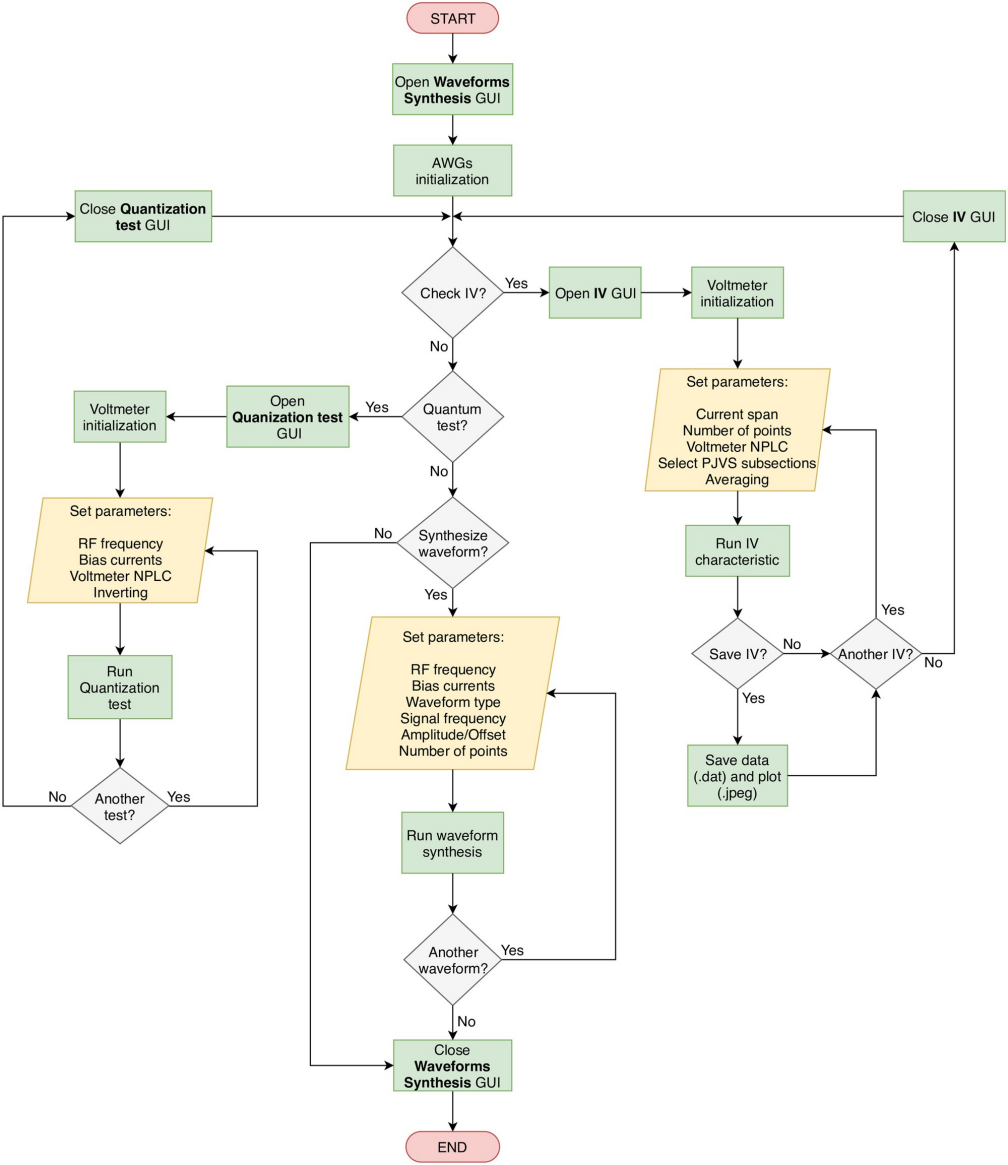
Flow chart of the Python software for the PJVS controlled operation.

The program execution follows the flow described by the chart in Fig 4.

The package is aimed at full customization including the Graphic User Interface (GUI), that consists of three main forms:

- PJVS Waveform Synthesis
- PJVS IV-characteristics
- PJVS Quantization Test

**Fig 5:**
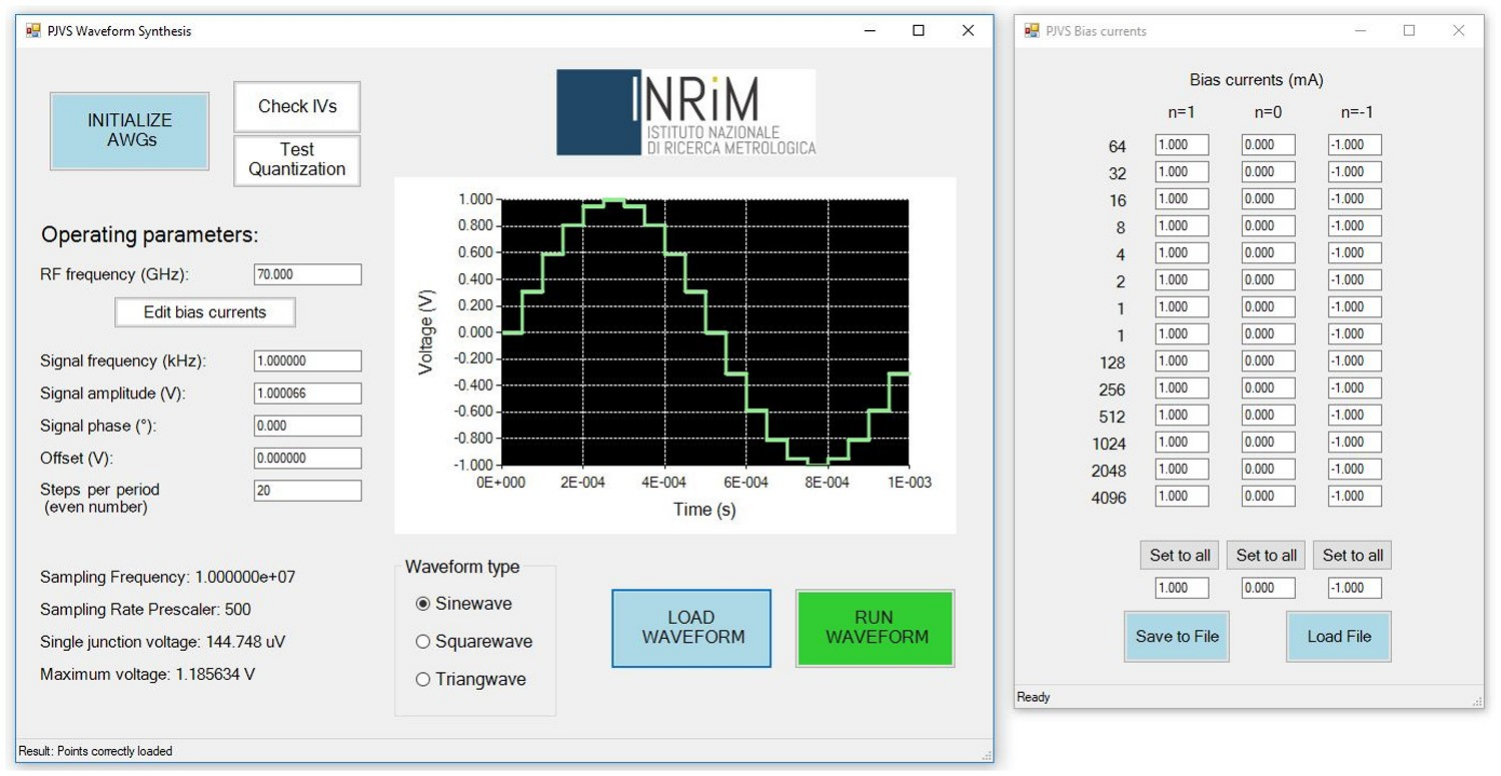
*PJVS Waveform Synthesis* GUI. The graph displays the expected output waveform. On the right, the form for setting, saving and loading bias currents is shown.

The *PJVS Waveform Synthesis* is the first GUI panel displayed (Fig 5). It provides for the initialization of the AWGs required for proper operation in the generation of quantum waveforms. We move its detailed description to the end of this section, since usually, a complete check of the PJVS behavior is required before proceeding with the waveforms synthesis. To that aim, two main tools are generally adopted, namely current-voltage characteristics and quantization test. Two dedicated forms are present in our software and both can be opened from the first one.

**Fig 6:**
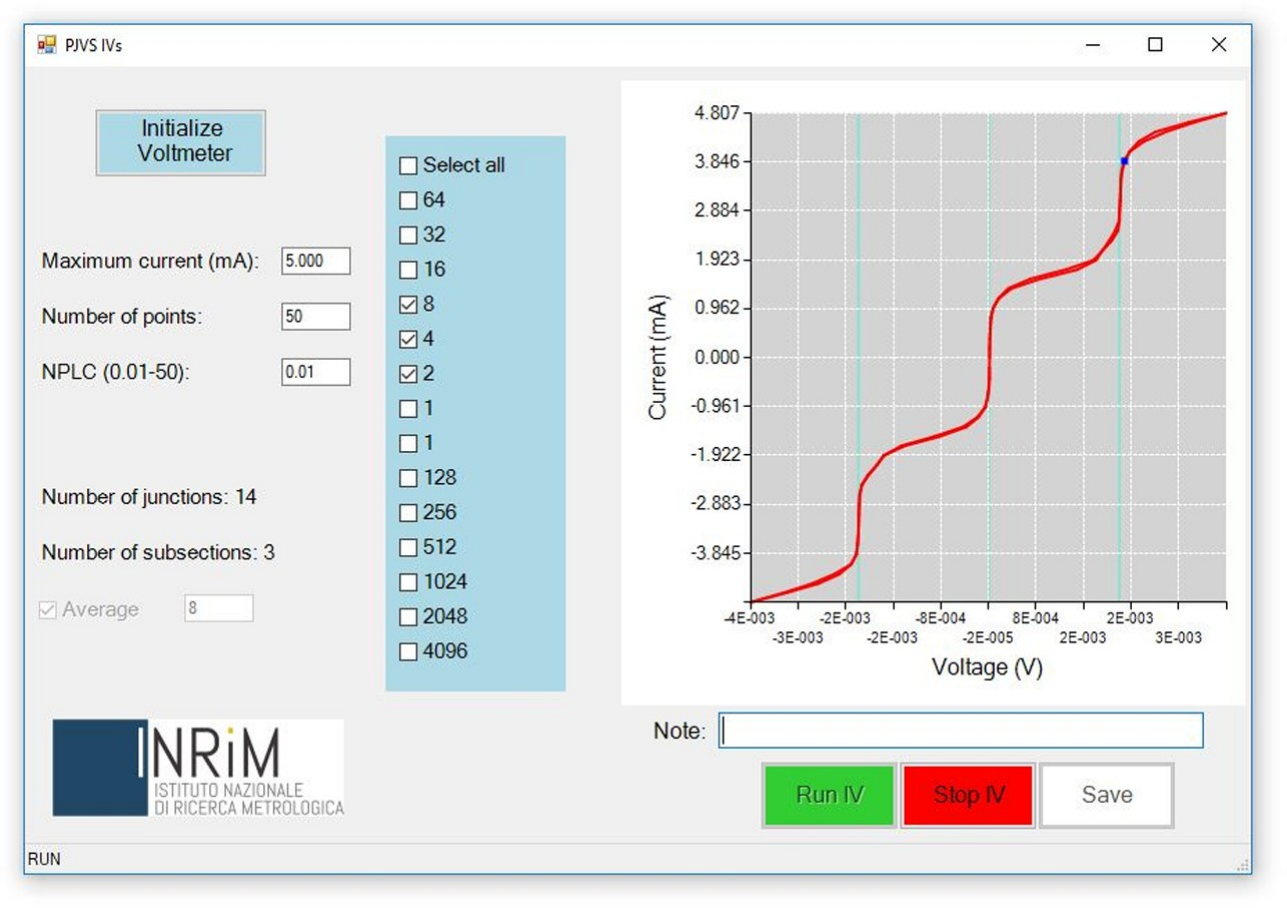
*PJVS IV-characteristics* GUI. The graph displays the averaged current-voltage characteristics of fourteen Josephson junctions subdivided in three consecutive sub-arrays.

After the proper initialization of the nanovoltmeter, the *PJVS IV-characteristics* form (Fig 6) allows the user to set few main parameters of the current-voltage characteristic to be displayed. These are current span, number of points and voltmeter number of power line cycles (NPLC). An interesting feature is the capability of separately checking current-voltage curves of individual subsections or a group of them. This is obtained by “flagging” specific checkboxes that set which sub-arrays have to be current-biased, with the constraint of being adjacent. In addition, the software allows to average an indefinite number of curves in order to get rid of possible background noise. Finally, voltage and current data points are displayed in a real-time graph that can be saved together with the data values.

**Fig 7:**
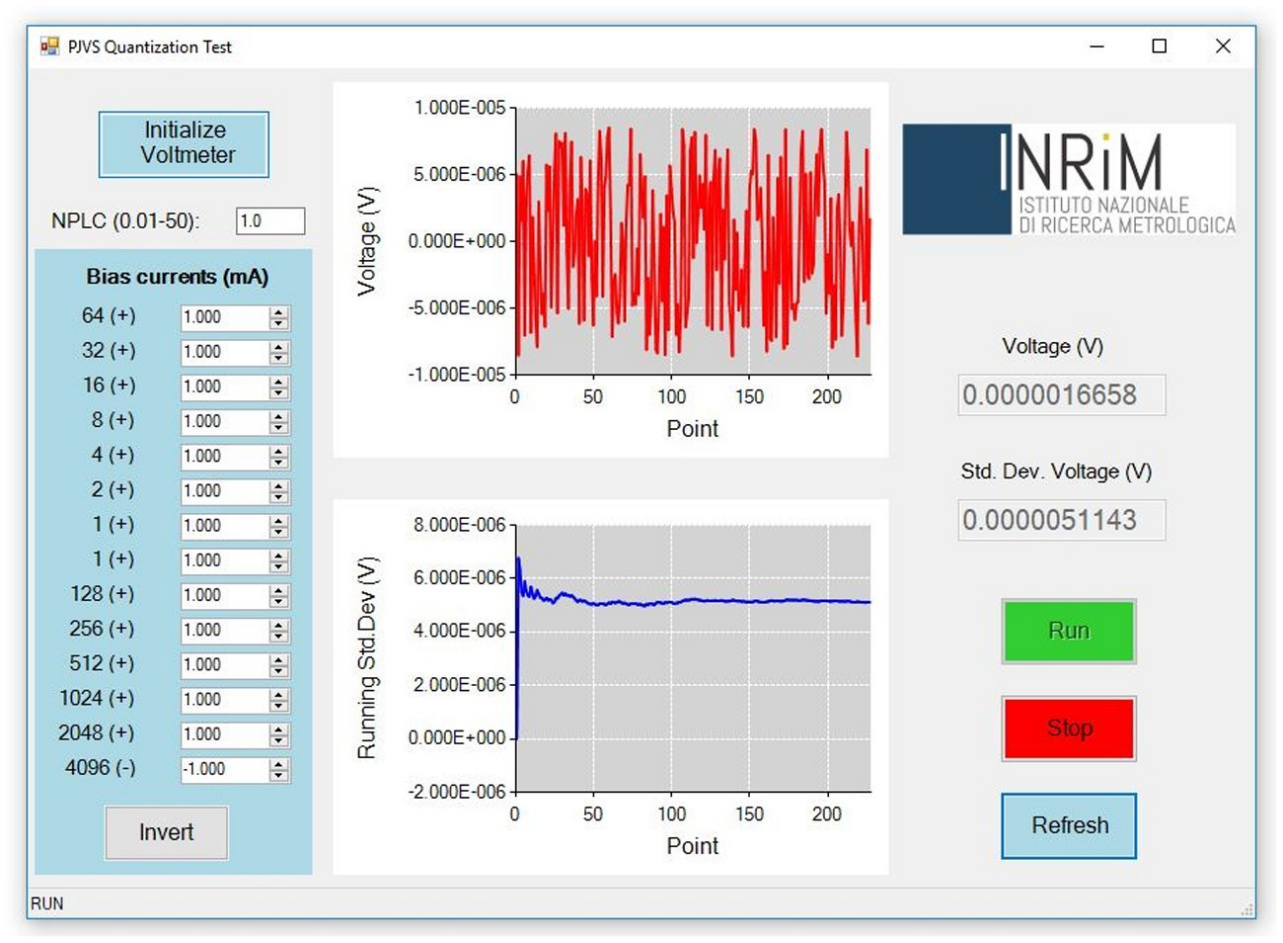
*PJVS Quantization Test* GUI. The two graphs display the voltage measurement and its running standard deviation.

The quantization test consists in measuring the voltage across the whole PJVS array when half of it (counting 4096 junctions) is biased on the *n* = +1 step and the second half on the *n* = −1 step (or vice versa), thus it is expected to be zero over a certain current range. The *PJVS Quantization Test* form (Fig 7) allows the user to perform this measurement and to vary the bias current of each subsection in order to determine the array operating margins, which are directly related to steps width. Two real-time graphs and textboxes help the user to monitor the PJVS output voltage measured by the nanovoltmeter and its running standard deviation.

After this checking phase, the user can go back to the *PJVS Waveform Synthesis* GUI for setting input parameters and the properties of the desired output waveforms. Input parameters are the frequency of the microwave excitation (*f*_*rf*_), necessary for the correct calculation of the voltage across each subsection, and bias currents for driving each sub-array on its *n* step. A dedicated form (right side of Fig 5) enables the user to set, save and load currents for each subsection and for each quantum step. The main output parameters are signal frequency, amplitude, offset, phase and number of points. After having successfully loaded the required voltage values on each AWG, the expected output waveform is displayed in the graph. At this point the system can be run and quantum voltage waveforms can be viewed and analyzed by any external oscilloscope or sampler.

### Developer’s point of view

The software is made up by four major functional blocks, all developed in Python. A short description of each module and of its main functions is presented below.

#### Math Functions.py

This script defines the functions to determine the AWGs output voltages for waveform synthesis and quantization test. Its main functions are:

- *EvalParameters*: calculates PJVS maximum output voltage and resolution via Eq 1.
- *CalcQuantumVolt*: converts a generic voltage into the closest quantized value obtainable with PJVS, determines which sub-arrays have to be switched on and calculates the required AWG voltages according to the electrical network analysis described in the last section of this paper.
- *CalcSine*, *CalcSquare* and *CalcTriang*: respectively sample ideal sine, square and triangular waves into a number of user-defined equally time-spaced points and quantize them via *CalcQuantumVolt* function.
- *QuantumTest*: calculates the AWG voltages required to bias one PJVS half on *n* = +1(−1) and the second half on *n* = −1(+1) quantum steps.

#### Devices Control.py

This script is responsible of the communication between the computer and the devices in use. It imports the interfacing libraries (GPIB-PyVISA [26] and AWG’s *dll*) and implements all remote-controlled operations (initialization, configuration, data I/O) of bias sources and voltmeter. Its main functions are:

- *InitAWGs*: initializes the AWGs and sets each channel as master or slave.
- *Load*: loads the calculated voltage samples for the waveform synthesis, sets the sampling frequency and the required output resistances for each AWG channel. Furthermore, it manages the trigger event between master and slaves.
- *Run* and *Stop*: respectively run and stop the generation of the AWGs loaded voltage samples.
- *InitVoltmeter*: initializes the voltmeter in use and sets its NPLC parameter.
- *SetOutputImpedance*: sets the required AWGs output impedances.
- *SetDCVoltages*: sets a dc voltage to each AWG channel given by a list of values passed as function arguments.

#### Graphics.py

This script is responsible of the creation of the GUIs and of their elements’ properties and functionalities. It is based on *MicroSoft Developer Network (MSDN) .NET* System.Windows.Forms [27] and System.Drawing [28] namespaces. It imports *Devices_Control.py* for linking any device functions (initialization, loading, etc.) to a graphic interactive element (button, textbox, checkbox, etc.). It imports *Math_Functions.py* for passing user input parameters as mathematical functions’ arguments. It performs and displays current-voltage characteristics following the procedure described in the last section of this paper.

#### Main.py

This script defines fundamental parameters that are used throughout the whole program, such array structure *N*_*J*_ and maximum AWG output voltage, and runs program execution by opening the main form.

## Further considerations and improvements

Thanks to its modularity, the program can be easily modified or integrated with new features by slightly revising few functional blocks. As an example, one can freely develop the GUIs adopting an alternative Python package, as *PyQt5*, *PyQt4*, *wxPython* or *Tk*. Furthermore, different instruments can be controlled by updating the main functions in *Devices_Control.py*. Indeed, interfacing with potentially any instrument is possible through the *PyVisa* GPIB library, or via USB with the *PyUSB* library, or by using directly the manufacturers *dll* libraries through *Python for .NET* [29]. As anticipated, fitting the software to different array structures is extremely straightforward, as it is accomplished just by varying one code line in *Main.py*.

As is well known, software packages are always improvable and continuously updated with new functionalities, and this is not an exception. Among the many possible enhancements, the most requested and useful feature for our package is the integration of a fast digital sampling voltmeter for a direct and real-time processing of the output quantum waveforms. This is of fundamental importance in the view of fully exploiting the high accuracy and reliability of Josephson arrays for ac voltage calibrations [12].

Integrating a temperature measurement and control system is another interesting upgrade, particularly useful when closed-cycle refrigeration is exploited [30, 31]. Josephson junctions electrical parameters are strictly dependent on temperature and its proper control is required to guarantee optimal operation.

## Insights on PJVS system settings and electrical network analysis

In the following, a detailed analysis of the electrical system is presented, by reference to the circuit in Fig 3. It is useful to better understand what the implemented main functions perform for calculating the output voltages *V*_*in*,*i*_ that properly drive PJVS.

### Waveform synthesis and quantization test system settings

When waveform synthesis is run, AWGs output resistances from *R*_1_ to *R*_14_ are set to 50 Ω, while *R*_0_ = 0 Ω (low impedance). As regards voltages, values from *V*_*in*,1_ to *V*_*in*,14_ have to be properly evaluated from the circuit Kirchhoff analysis, while *V*_0_ = 0 V. This means that subsection “64” is grounded. The reason why a dedicated channel is employed for this grounded connection will be apparent when current-voltage curves implementation is discussed in the next section.

Knowing the voltage across the *i*^*th*^ sub-array *V*_*J*,*i*,*n*_ = *nN*_*J*_(*i*)*hf*_*rf*_/2*e*, the current *I*_*J*,*i*,*n*_ for biasing it on *n* = 0 or ±1 quantum step and applying Kirchhoff rules we get:

$$\begin{array}{c} \begin{array}{cc} & V_{in,1}=V_{J,1,n}+R_{1}\left(I_{J,1,n}-I_{J,2,n}\right)\\ & V_{in,2}=V_{J,1,n}+V_{J,2,n}+R_{2}\left(I_{J,2,n}-I_{J,3,n}\right)\\ & V_{in,3}=V_{J,1,n}+V_{J,2,n}+V_{J,3,n}+R_{3}\left(I_{J,3,n}-I_{J,4,n}\right)\\ & \ldots \ldots \\ & V_{in,14}=V_{J,1,n}+V_{J,2,n}+V_{J,3,n}+\ldots .+V_{J,13,n}+V_{J,14,n}+R_{14}I_{J,14,n} \end{array} \end{array}$$ (2)

where currents *I*_*J*,*i*,*n*_ are taken as positive when circulating from *V*_*out*,+_ to *V*_*out*,−_ according to Fig 3.

A particular case occurs when quantization test is carried out: here *n* = +1(−1) for *i* = 1 to 13, whereas *n* = −1(+1) for *i* = 14.

### Current-voltage characteristics

As previously described, current-voltage curves of one or more consecutive subsections can be performed and visualized. This is achieved by properly setting output resistance and voltage of each AWG channel. To current-bias consecutive subsections from *j* to *k*, with 1 ≤ *j* ≤ 14 and *j* ≤ *k* ≤ 14, output resistances and voltages are to be set as follow:

$$\begin{array}{c} \begin{array}{cc} & R_{j-1}=0\;\Omega \\ & R_{k}=50\;\Omega ,\\ & R_{i}=\infty \text{,}\;\text{for}\;i\neq j-1\;\text{and}\;i\neq k\\ & V_{in,i}=0\;\text{V},\;\text{for}\;i\neq k \end{array} \end{array}$$ (3)

Therefore, the current *I*_*J*,*j*÷*k*_ flowing into the selected subsections is given by

$$\begin{array}{c} I_{J,j÷k}=\frac{V_{out}-V_{in,k}}{R_{k}} \end{array}$$ (4)

and a *I*_*J*,*j*÷*k*_ to *V*_*out*_ curve is obtained for different *V*_*in*,*k*_ values. As can be seen, only two channels at time are used since all the others are set to high-impedance.

## Conclusion

An open-source modular and easily customizable Python package for PJVS operation and control was presented. The software allows the synthesis of quantum-based voltage waveforms through automated control of four AWGs, the check of sub-arrays correct operation via current-voltage characteristics and quantization tests by means of a nanovoltmeter. The package can be fully reconfigured and readily expanded to suit any practical needs, such as the replacement of the setup instruments or the integration of additional tests and functions.

The software, shared on *GitHub* [22], is aimed at being developed and tested in cooperation with all interested NMIs. It can also be run in simulation mode, allowing the interested user to directly check its features without the need of a real experimental setup.
